# Supplementary material for: Small extracellular vesicles from human bone marrow mesenchymal stromal cells enhance migration and regulate reparative gene expression in dermal fibroblasts
Source: Sci Rep. 2025 Jun 3;15:19383. doi: 10.1038/s41598-025-04057-6 (PMC12134064; doi:10.1038/s41598-025-04057-6)
Supplement: Supplementary file 1 — Supplementary Material 1 [file 41598_2025_4057_MOESM1_ESM.pdf]

Supplementary Information

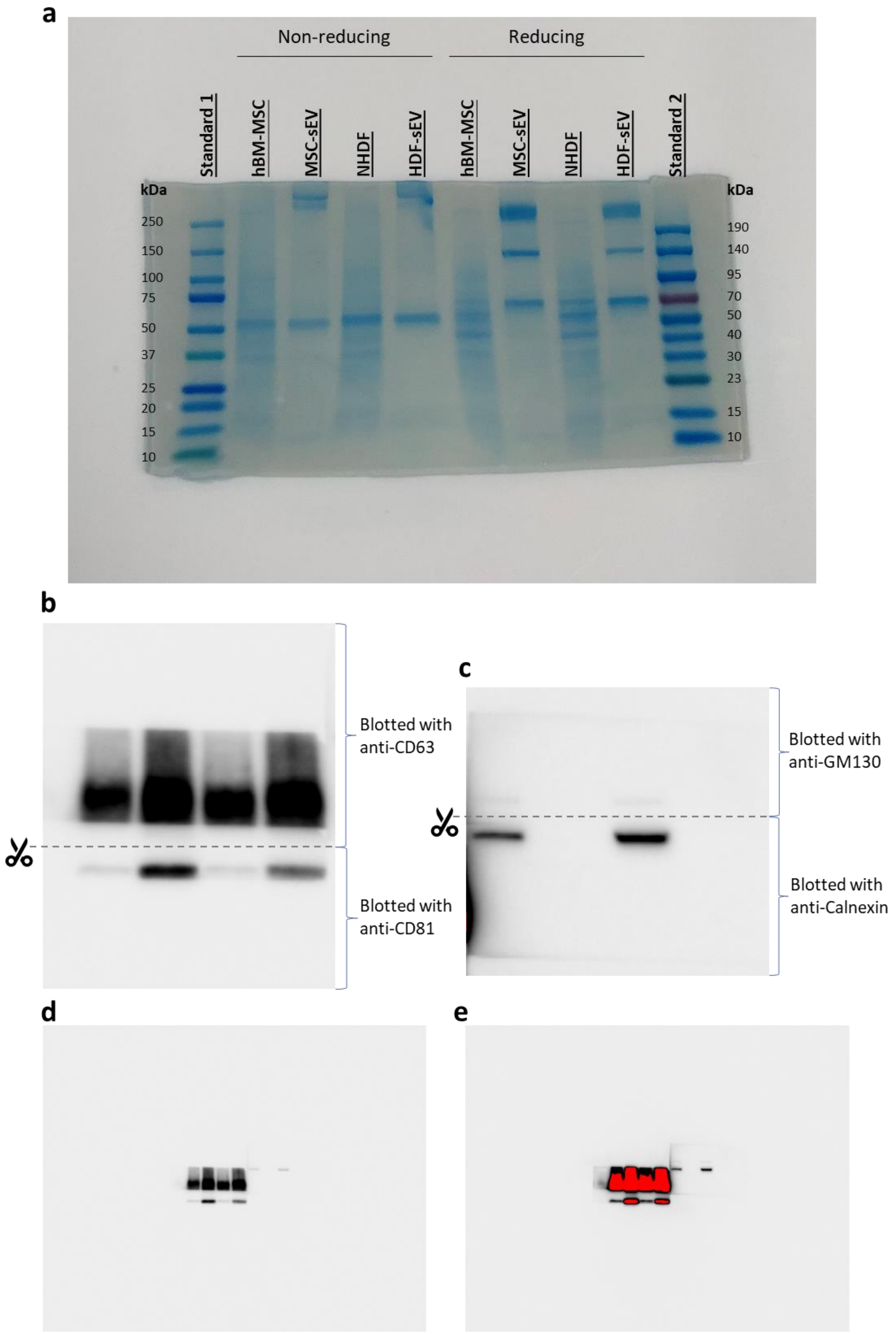

Supplementary Figure 1. Original images from the western blot data shown in Figure 2d. (a) Proteins in the gel visualised by Coomassie Blue. Anti-CD63 antibody and anti-CD81 antibody should be used under non-reducing condition (reducing agent was not added to the sample). Anti-GM130 antibody and anti-Calnexin antibody should be used under reducing condition. (b) Western blot for CD63 and CD81. (c) Western blot for GM130 and Calnexin. (d) The original uncropped images of (b). (e) The original uncropped images of (c).

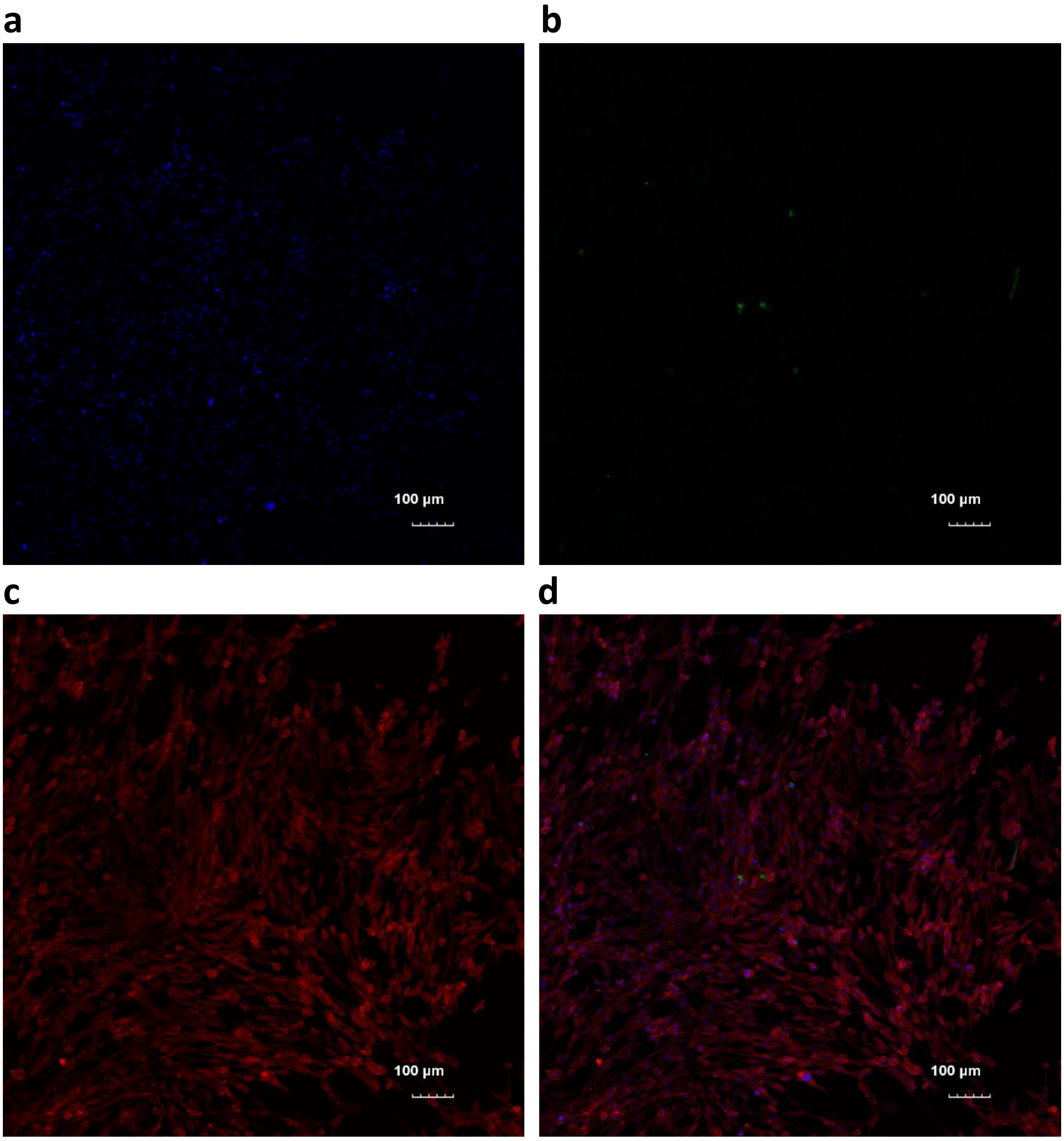

Supplementary Figure 2. Wide field confocal fluorescence imaging (includes over 100 cells) of MSC-sEV uptake by NHDFs, providing a representative field of uptake distribution. (a) Blue channel: DAPI (nuclei). (b) Green channel: PKH67-labeled sEVs were found from multiple green signals. (c) Red channel: Phalloidin (F-actin). (d) Merged image for all fluorescence channels.

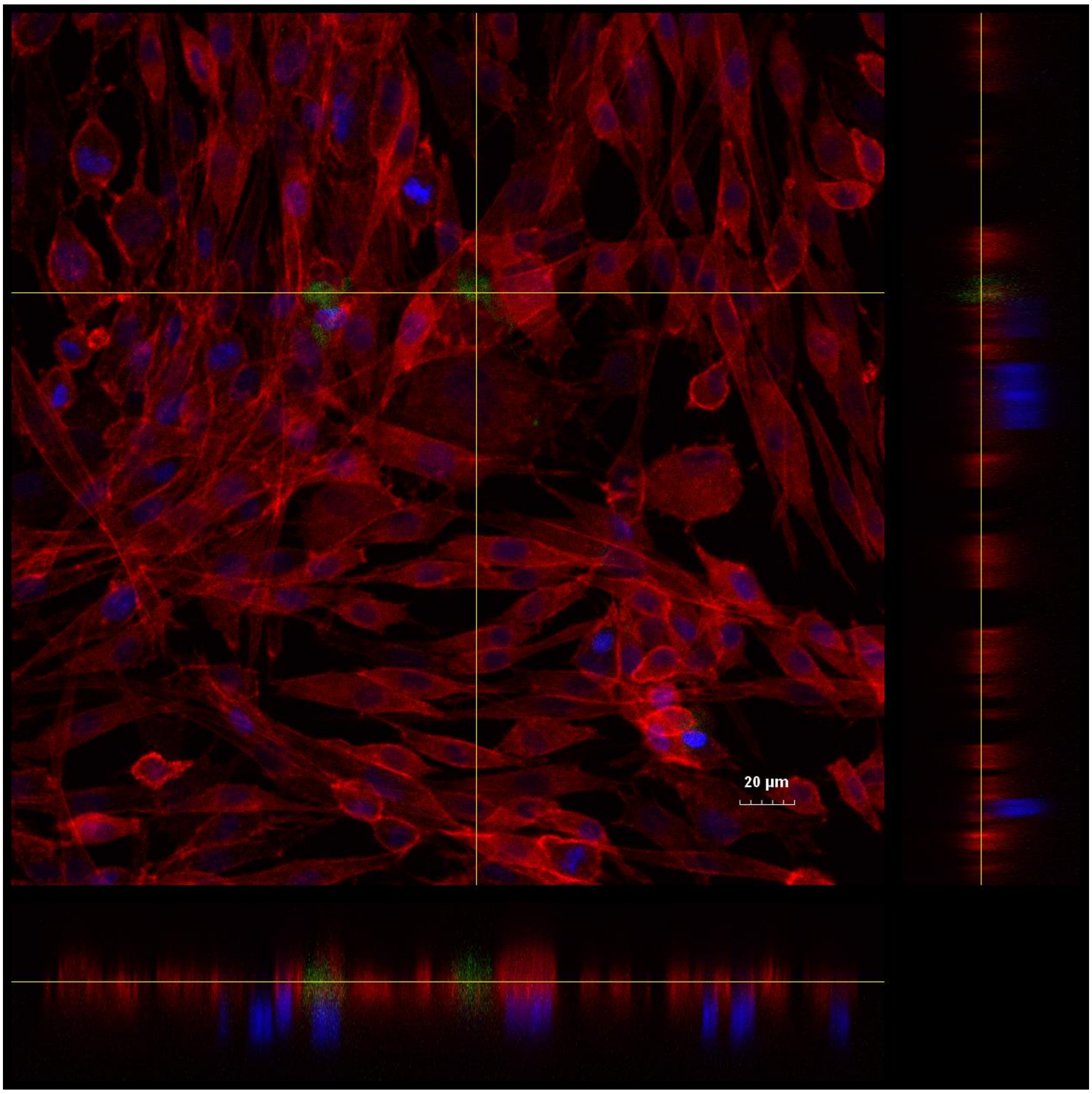

Supplementary Figure 3. Z-stack reconstruction confirming intracellular uptake of MSC-sEVs. Orthogonal view of confocal Z-stacks shows PKH67-labeled EVs localized within the NHDFs (defined by phalloidin stain). Green: PKH67-labeled sEVs, Red: Phalloidin (F-actin), Blue: DAPI (nuclei).

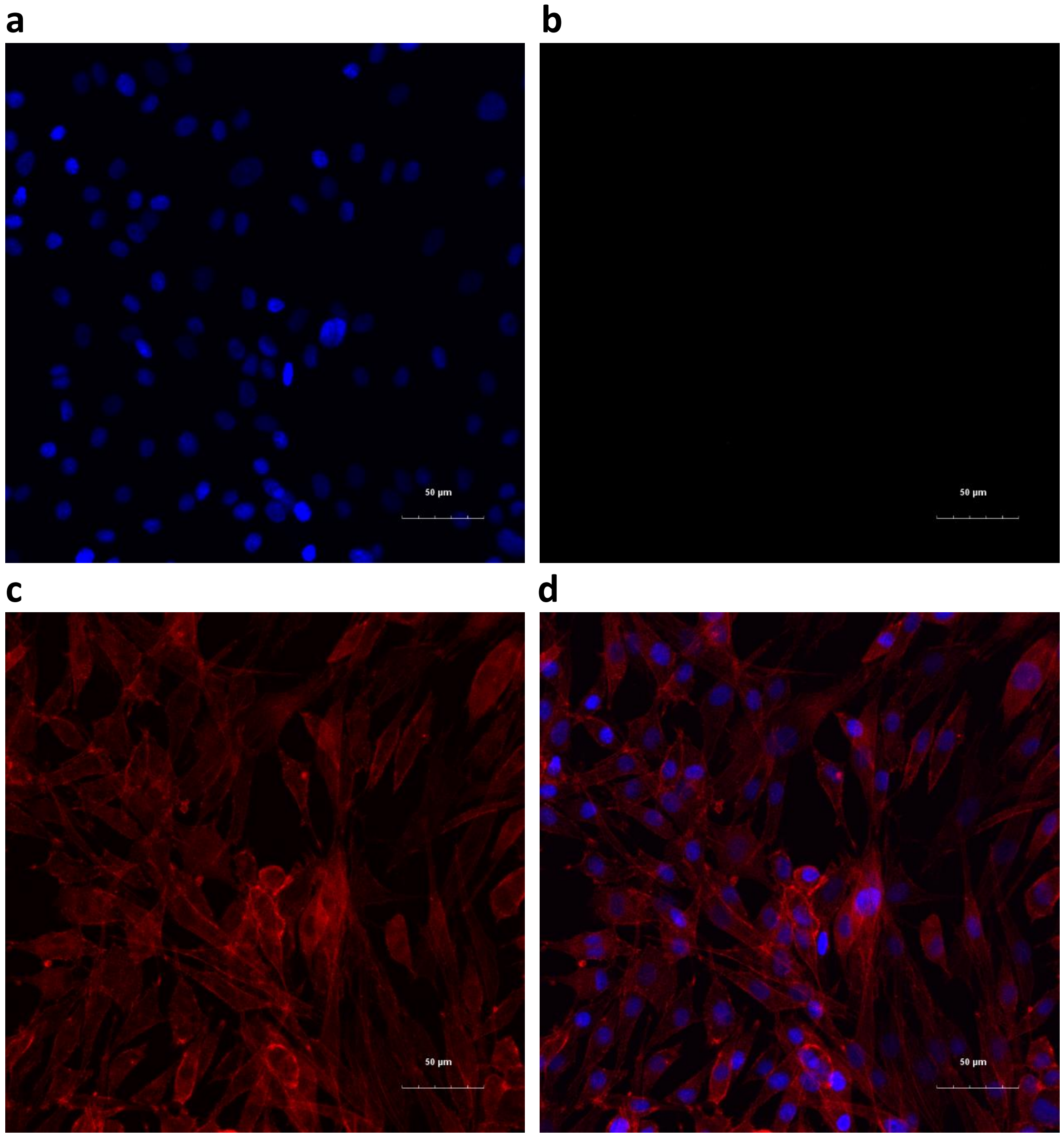

Supplementary Figure 4. A PKH67-only control (no EVs) confirms minimal background fluorescence and rules out nonspecific staining. (a) Blue channel: DAPI (nuclei). (b) Green channel: no green signal was found. (c) Red channel: Phalloidin (F-actin). (d) Merged image for all fluorescence channels.

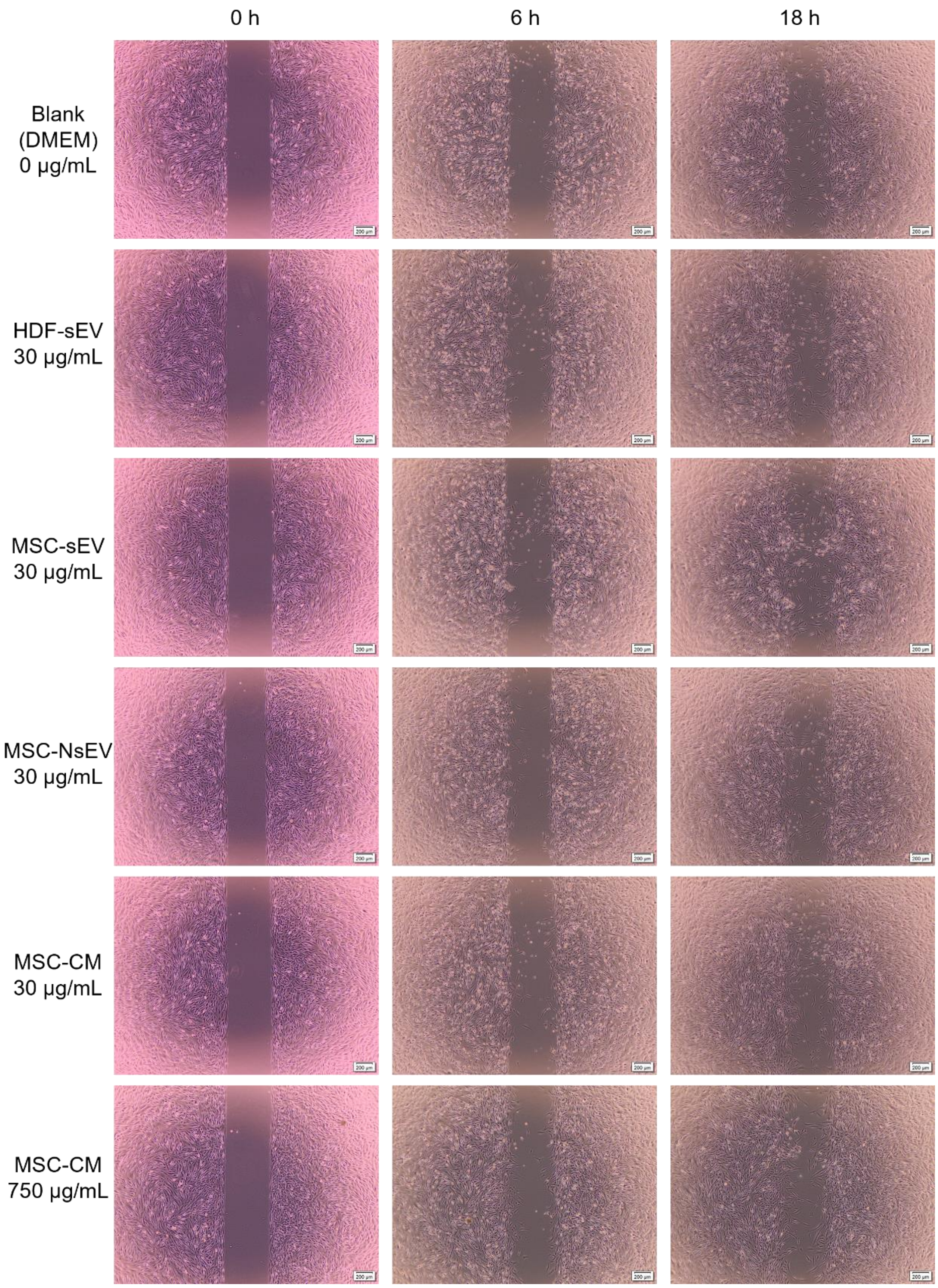

Supplementary Figure 5. Bright field images of NHDFs treated with different secretome fractions at 0 h, 6 h and 18 h. The migration experiment was repeated three times using independent biological samples. In each experiment, triplicates were performed for each condition.

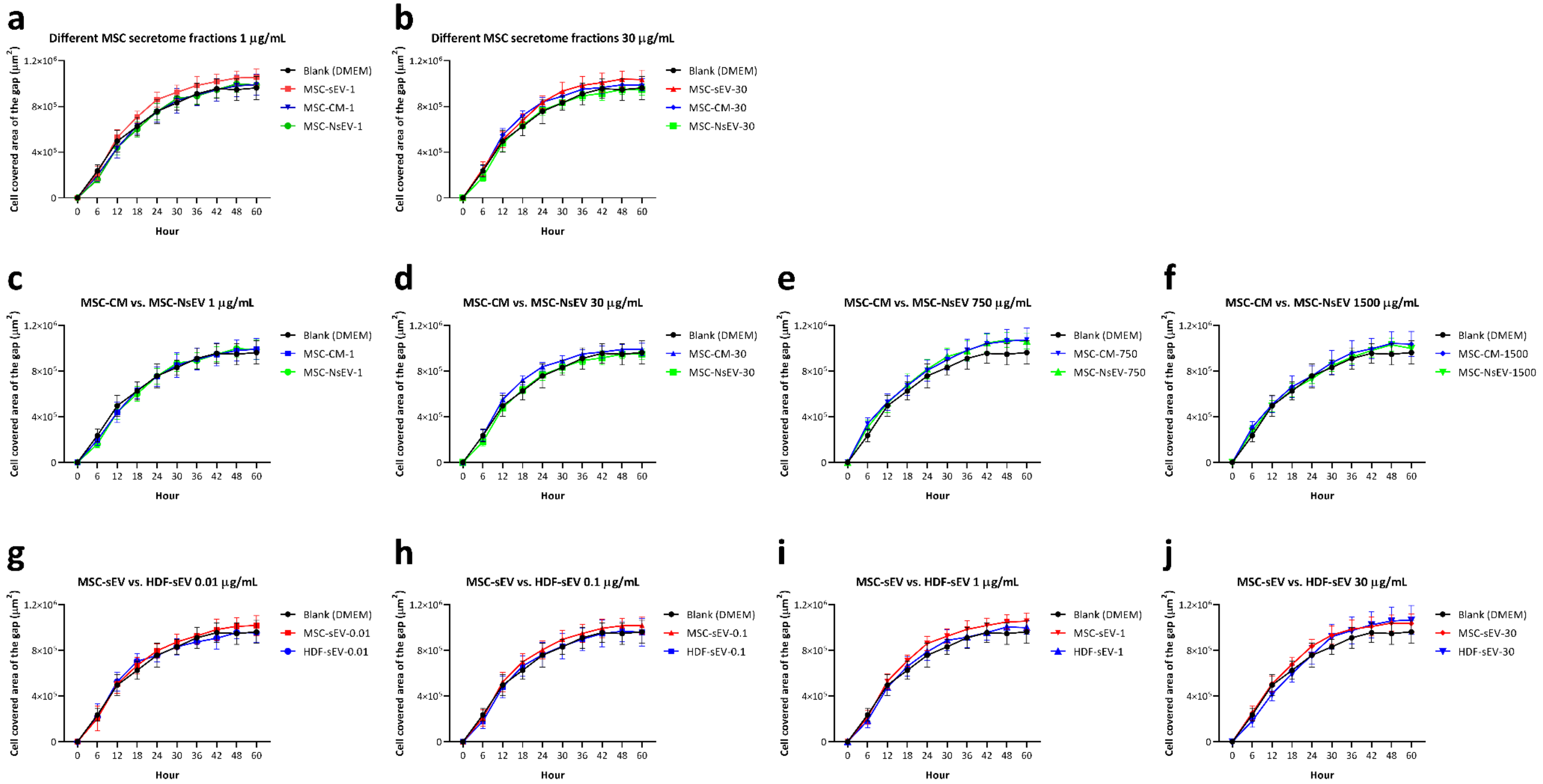

Supplementary Figure 6. The migration curves of the NHDFs treated by different secretome fractions at different concentrations. Cell-covered area over time was quantified during the NHDF migration assay. (a, b) NHDF migration in response to MSC-sEV, MSC-CM, and MSC-NsEV at 1 µg/mL and 30 µg/mL, respectively. (c–f) Comparison of MSC-CM and MSC-NsEV at increasing concentrations (1 µg/mL, 30 µg/mL, 750 µg/mL, and 1500 µg/mL). (g–j) Comparison

of MSC-sEV and HDF-sEV at different concentrations (0.01 µg/mL, 0.1 µg/mL, 1 µg/mL, and 30 µg/mL). Data are presented as mean ± SD from three independent biological replicates, with each condition performed in triplicate.

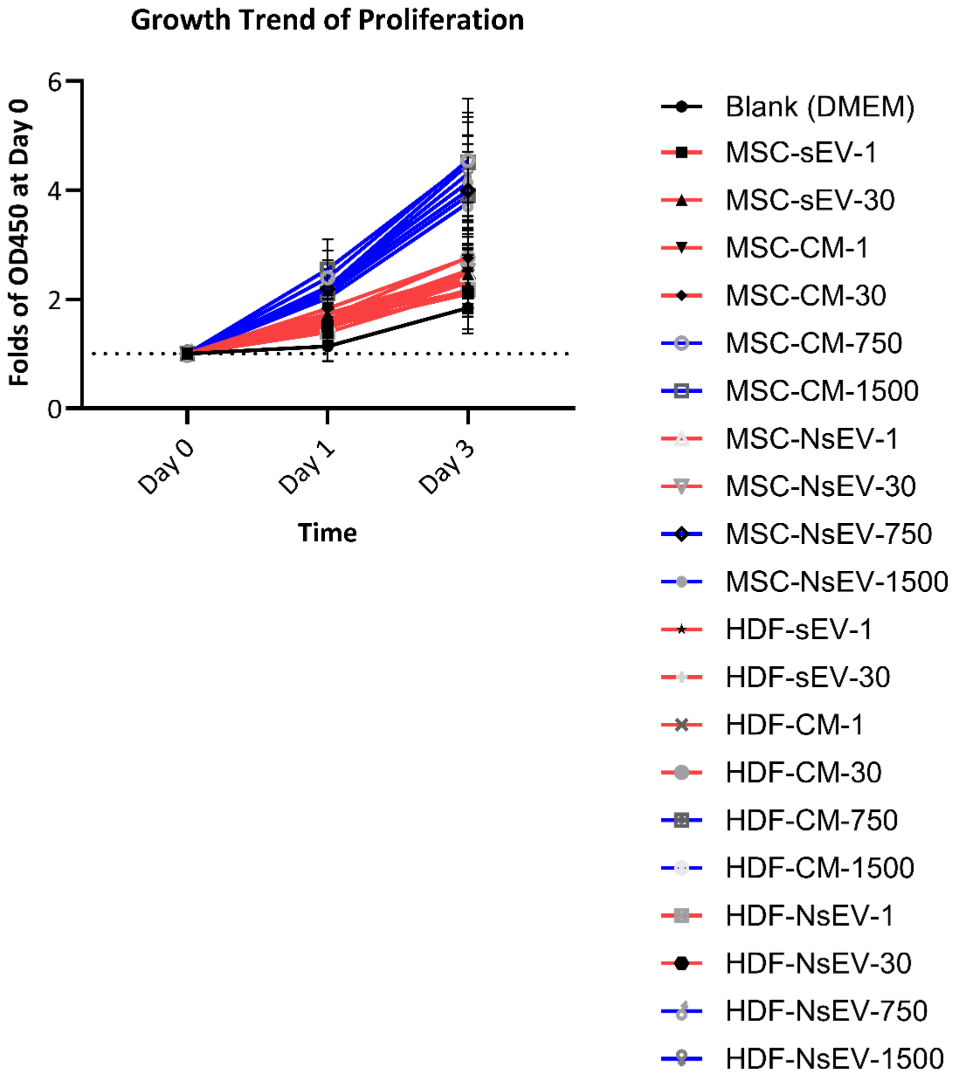

Supplementary Figure 7. The proliferation curves of the NHDFs treated by different secretome fractions at different concentrations. MSC-sEV at 1 µg/mL and 30 µg/mL, MSC-CM and MSC-NsEV at 1 µg/mL, 30 µg/mL, 750 µg/mL, and 1500 µg/mL, respectively. Data are presented as mean ± SD from three independent biological replicates, with each condition performed in triplicate.

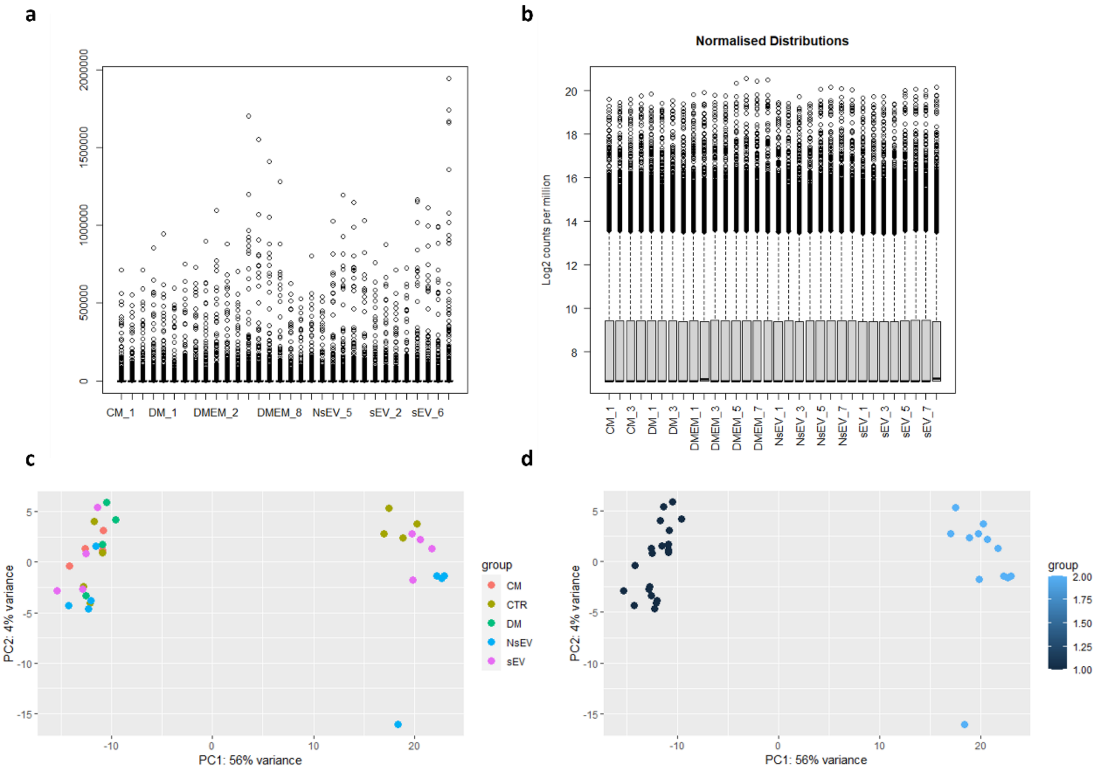

Supplementary Figure 8. Quality control of reads and Principal Component Analysis (PCA). (a) Box plots displaying distribution of reads between samples. (b) Normalised distribution of reads. (c) PCA displaying major sources of variance between samples, according to their treatment. (d) PCA displaying major sources of variance between samples according to the batch, attesting for a batch effect.

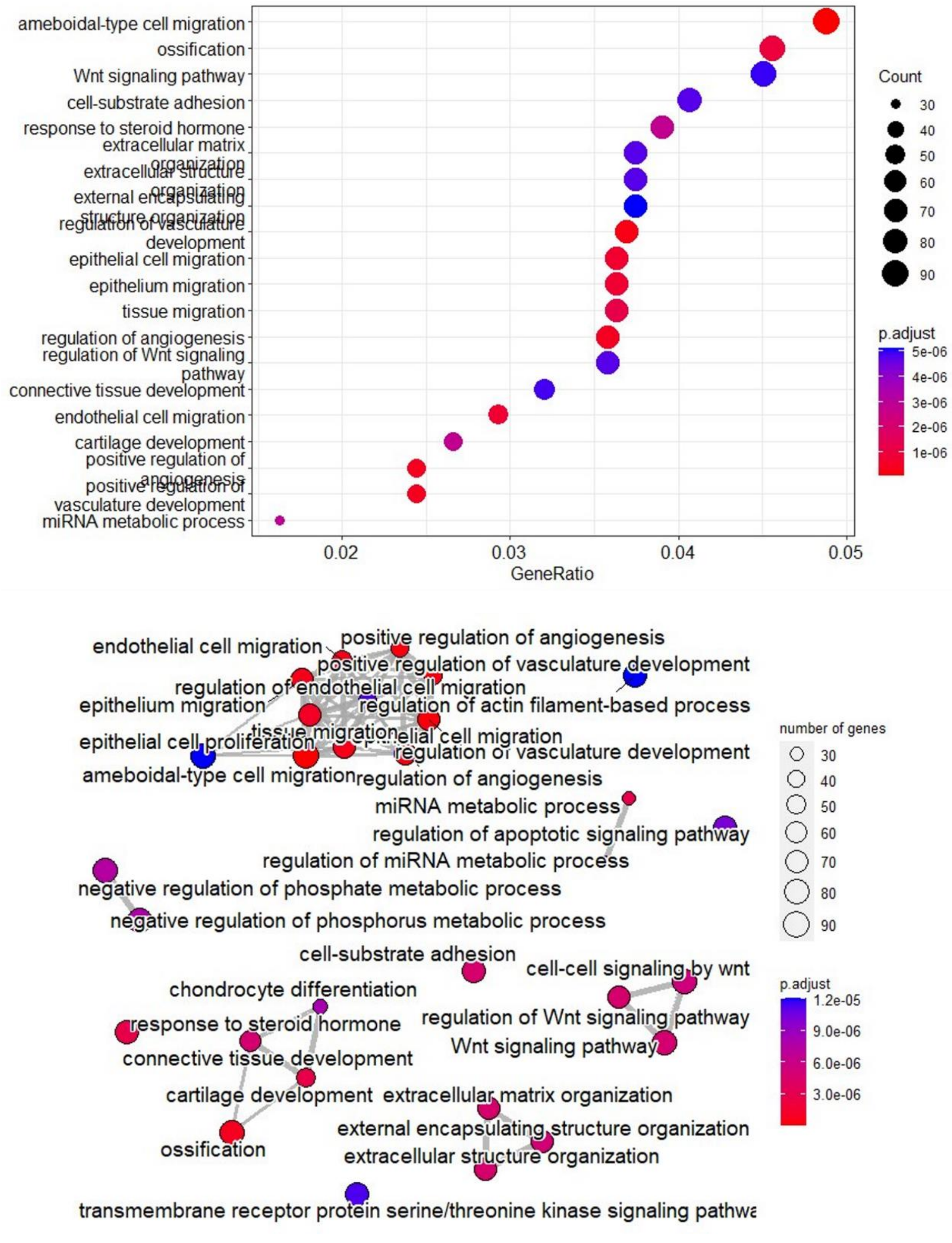

Supplementary Figure 9. Gene Ontology Analysis and Enriched Pathways Clustering when comparing samples exposed to NsEVs and controls. (a) Pathways that have been significantly changed based on the number of genes within them that are differentially expressed. (b) Links between enriched pathways and how they cluster depending on common genes.

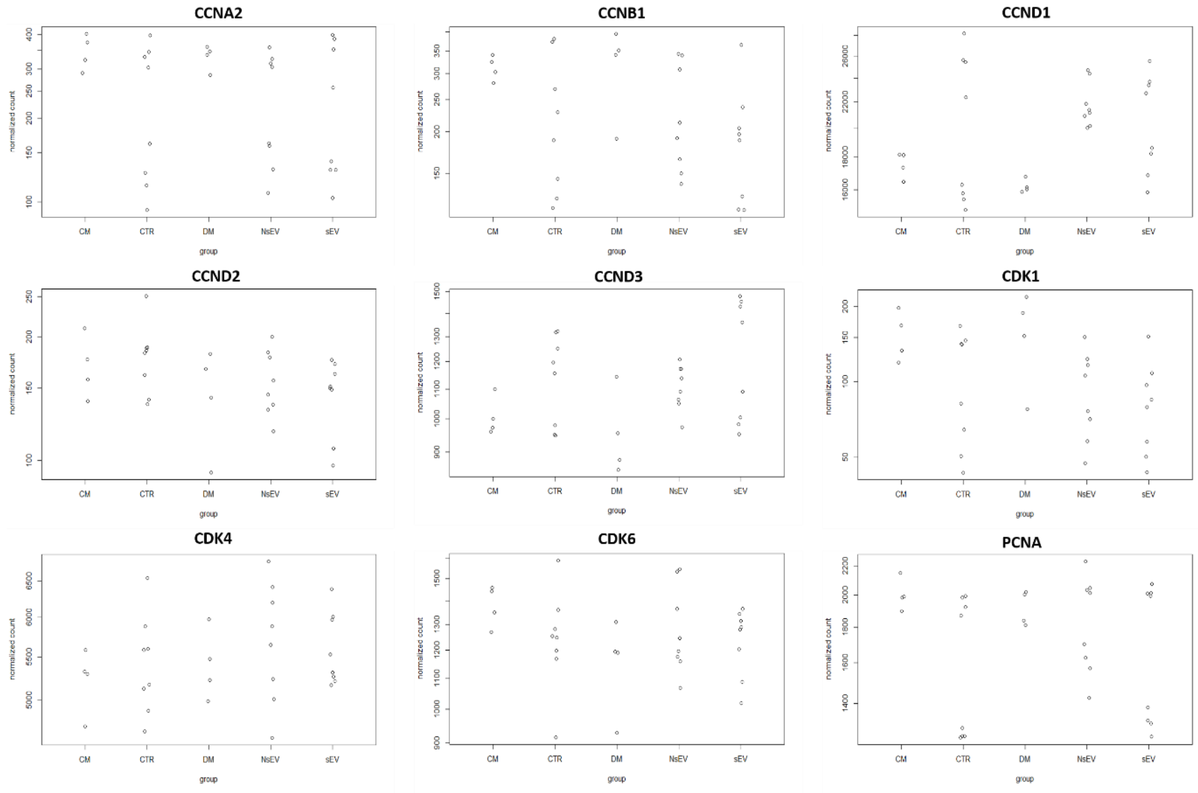

Supplementary Figure 10. Normalised Counts of cell cycle markers Cyclin A2, Cyclin B1, Cyclin D1, Cyclin D2, Cyclin D3, CDK1, CDK4, CDK6 and PCNA. Expression was not significantly different between conditions. Differential gene expression analysis was performed using DESeq2. Statistical significance was determined using the Wald test, and p-values were adjusted for multiple comparisons using the Benjamini-Hochberg method (adjusted p-value < 0.05 was considered significant).

Supplementary Table 1 GLM ANOVA results for migration (cell covered area of the gap in μm2) of NHDFs in response to factors of migration time (Hour), three secretome fraction types from MSC (Treatment) and concentration of secretome (Concentration). DF is the abbreviation for Degree of Freedom.

Variable	DF	F Value	P Value
Hour	9	2032.78	<0.001
Treatment	2	29.77	<0.001
Concentration	2	11.48	<0.001
Hour*Treatment	18	0.62	0.889
Hour*Concentration	18	2.10	0.005
Treatment*Concentration	4	11.38	<0.001

Supplementary Table 2 Results of Tukey's post-hoc multiple comparisons between different secretome fractions of MSC at different concentrations (e.g. MSC-sEV-1 means the group treated with MSC-sEV at 1 μg/mL) for their effects on migration behaviour of NHDFs. Mean Diff. is the abbreviation for Mean Difference.

Tukey's multiple comparisons test	Mean 1	Mean2	Mean Diff.	Significance	P Value
Blank (DMEM) vs. MSC-sEV-1	672165	734542	-62376	****	<0.0001
Blank (DMEM) vs. MSC-sEV-30	672165	728205	-56040	****	<0.0001
Blank (DMEM) vs. MSC-CM-1	672165	668993	3172	ns	>0.9999
Blank (DMEM) vs. MSC-CM-30	672165	713417	-41251	***	0.0007
Blank (DMEM) vs. MSC-NsEV-1	672165	665273	6893	ns	0.9927
Blank (DMEM) vs. MSC-NsEV-30	672165	659940	12225	ns	0.8787
MSC-sEV-1 vs. MSC-sEV-30	734542	728205	6336	ns	0.9954
MSC-sEV-1 vs. MSC-CM-1	734542	668993	65548	****	<0.0001
MSC-sEV-1 vs. MSC-CM-30	734542	713417	21125	ns	0.3304
MSC-sEV-1 vs. MSC-NsEV-1	734542	665273	69269	****	<0.0001
MSC-sEV-1 vs. MSC-NsEV-30	734542	659940	74602	****	<0.0001
MSC-sEV-30 vs. MSC-CM-1	728205	668993	59212	****	<0.0001
MSC-sEV-30 vs. MSC-CM-30	728205	713417	14789	ns	0.7458
MSC-sEV-30 vs. MSC-NsEV-1	728205	665273	62933	****	<0.0001
MSC-sEV-30 vs. MSC-NsEV-30	728205	659940	68265	****	<0.0001
MSC-CM-1 vs. MSC-CM-30	668993	713417	-44423	***	0.0002
MSC-CM-1 vs. MSC-NsEV-1	668993	665273	3720	ns	0.9998
MSC-CM-1 vs. MSC-NsEV-30	668993	659940	9053	ns	0.9698
MSC-CM-30 vs. MSC-NsEV-1	713417	665273	48144	****	<0.0001
MSC-CM-30 vs. MSC-NsEV-30	713417	659940	53477	****	<0.0001
MSC-NsEV-1 vs. MSC-NsEV-30	665273	659940	5333	ns	0.9982

Supplementary Table 3 GLM ANOVA results for migration (cell covered area of the gap in μm2) of NHDFs in response to factors of migration time (Hour), two secretome fraction types from MSC (Treatment) and concentration of secretome (Concentration). DF is the abbreviation for Degree of Freedom.

Variable	DF	F Value	P Value
Hour	9	2143.94	<0.001
Treatment	1	11.20	0.001
Concentration	4	31.06	<0.001
Hour*Treatment	9	0.43	0.919
Hour*Concentration	36	2.20	<0.001
Treatment*Concentration	4	4.75	0.001

Supplementary Table 4 Results of Tukey's post-hoc multiple comparisons between two secretome fractions of MSC at different concentrations (e.g. MSC-CM-1 means the group treated with MSC-CM at 1 µg/mL) for their effects on migration behaviour of NHDFs. Mean Diff. is the abbreviation for Mean Difference.

Tukey's multiple comparisons test	Mean 1	Mean2	Mean Diff.	Significance	P Value
Blank (DMEM) vs. MSC-CM-1	672165	668993	3172	ns	>0.9999
Blank (DMEM) vs. MSC-CM-30	672165	713417	-41251	**	0.0035
Blank (DMEM) vs. MSC-CM-750	672165	740091	-67926	****	<0.0001
Blank (DMEM) vs. MSC-CM-1500	672165	714119	-41953	**	0.0027
Blank (DMEM) vs. MSC-NsEV-1	672165	665273	6893	ns	0.9993
Blank (DMEM) vs. MSC-NsEV-30	672165	659940	12225	ns	0.9656
Blank (DMEM) vs. MSC-NsEV-750	672165	740701	-68535	****	<0.0001
Blank (DMEM) vs. MSC-NsEV-1500	672165	691481	-19315	ns	0.6674
MSC-CM-1 vs. MSC-CM-30	668993	713417	-44423	**	0.0010
MSC-CM-1 vs. MSC-CM-750	668993	740091	-71098	****	<0.0001
MSC-CM-1 vs. MSC-CM-1500	668993	714119	-45125	***	0.0008
MSC-CM-1 vs. MSC-NsEV-1	668993	665273	3720	ns	>0.9999
MSC-CM-1 vs. MSC-NsEV-30	668993	659940	9053	ns	0.9951
MSC-CM-1 vs. MSC-NsEV-750	668993	740701	-71707	****	<0.0001
MSC-CM-1 vs. MSC-NsEV-1500	668993	691481	-22487	ns	0.4593
MSC-CM-30 vs. MSC-CM-750	713417	740091	-26674	ns	0.2257
MSC-CM-30 vs. MSC-CM-1500	713417	714119	-702	ns	>0.9999
MSC-CM-30 vs. MSC-NsEV-1	713417	665273	48144	***	0.0002
MSC-CM-30 vs. MSC-NsEV-30	713417	659940	53477	****	<0.0001
MSC-CM-30 vs. MSC-NsEV-750	713417	740701	-27284	ns	0.1994
MSC-CM-30 vs. MSC-NsEV-1500	713417	691481	21936	ns	0.4952
MSC-CM-750 vs. MSC-CM-1500	740091	714119	25972	ns	0.2586
MSC-CM-750 vs. MSC-NsEV-1	740091	665273	74818	****	<0.0001
MSC-CM-750 vs. MSC-NsEV-30	740091	659940	80151	****	<0.0001
MSC-CM-750 vs. MSC-NsEV-750	740091	740701	-609.5	ns	>0.9999
MSC-CM-750 vs. MSC-NsEV-1500	740091	691481	48610	***	0.0002
MSC-CM-1500 vs. MSC-NsEV-1	714119	665273	48846	***	0.0002
MSC-CM-1500 vs. MSC-NsEV-30	714119	659940	54179	****	<0.0001
MSC-CM-1500 vs. MSC-NsEV-750	714119	740701	-26582	ns	0.2298
MSC-CM-1500 vs. MSC-NsEV-1500	714119	691481	22638	ns	0.4497
MSC-NsEV-1 vs. MSC-NsEV-30	665273	659940	5333	ns	0.9999
MSC-NsEV-1 vs. MSC-NsEV-750	665273	740701	-75428	****	<0.0001
MSC-NsEV-1 vs. MSC-NsEV-1500	665273	691481	-26208	ns	0.2472
MSC-NsEV-30 vs. MSC-NsEV-750	659940	740701	-80761	****	<0.0001
MSC-NsEV-30 vs. MSC-NsEV-1500	659940	691481	-31541	ns	0.0736
MSC-NsEV-750 vs. MSC-NsEV-1500	740701	691481	49220	***	0.0001

Supplementary Table 5 GLM ANOVA results for migration (cell covered area of the gap in μm2) of NHDFs in response to factors of migration time (Hour), cell source of sEVs (sEV type) and concentration of sEVs (Concentration). DF is the abbreviation for Degree of Freedom.

Variable	DF	F Value	P Value
Hour	9	1844.09	<0.001
sEV type	1	30.40	<0.001
Concentration	4	9.28	<0.001
Hour*sEV type	9	0.53	0.850
Hour*Concentration	36	1.70	0.007
sEV type*Concentration	4	2.49	0.042

Supplementary Table 6 Results of Tukey's post-hoc multiple comparisons between different sEV types at different concentrations (e.g. MSC-sEV-0.01 means the group treated with MSC-sEV at 0.01 μg/mL) for their effects on migration behaviour of NHDFs. Mean Diff. is the abbreviation for Mean Difference.

Tukey's multiple comparisons test	Mean 1	Mean2	Mean Diff.	Significance	P Value
Blank (DMEM) vs. MSC-sEV-0.01	672165	698860	-26695	ns	0.3469
Blank (DMEM) vs. MSC-sEV-0.1	672165	711355	-39189	*	0.0225
Blank (DMEM) vs. MSC-sEV-1	672165	734542	-62376	****	<0.0001
Blank (DMEM) vs. MSC-sEV-30	672165	728205	-56040	****	<0.0001
Blank (DMEM) vs. HDF-sEV-0.01	672165	672552	-386.9	ns	>0.9999
Blank (DMEM) vs. HDF-sEV-0.1	672165	669409	2756	ns	>0.9999
Blank (DMEM) vs. HDF-sEV-1	672165	687378	-15212	ns	0.9291
Blank (DMEM) vs. HDF-sEV-30	672165	700293	-28127	ns	0.2759
MSC-sEV-0.01 vs. MSC-sEV-0.1	698860	711355	-12494	ns	0.9778
MSC-sEV-0.01 vs. MSC-sEV-1	698860	734542	-35681	ns	0.0569
MSC-sEV-0.01 vs. MSC-sEV-30	698860	728205	-29345	ns	0.2230
MSC-sEV-0.01 vs. HDF-sEV-0.01	698860	672552	26308	ns	0.3675
MSC-sEV-0.01 vs. HDF-sEV-0.1	698860	669409	29451	ns	0.2187
MSC-sEV-0.01 vs. HDF-sEV-1	698860	687378	11483	ns	0.9870
MSC-sEV-0.01 vs. HDF-sEV-30	698860	700293	-1432	ns	>0.9999
MSC-sEV-0.1 vs. MSC-sEV-1	711355	734542	-23187	ns	0.5489
MSC-sEV-0.1 vs. MSC-sEV-30	711355	728205	-16851	ns	0.8785
MSC-sEV-0.1 vs. HDF-sEV-0.01	711355	672552	38802	*	0.0251
MSC-sEV-0.1 vs. HDF-sEV-0.1	711355	669409	41945	*	0.0100
MSC-sEV-0.1 vs. HDF-sEV-1	711355	687378	23977	ns	0.5013
MSC-sEV-0.1 vs. HDF-sEV-30	711355	700293	11062	ns	0.9898
MSC-sEV-1 vs. MSC-sEV-30	734542	728205	6336	ns	0.9998
MSC-sEV-1 vs. HDF-sEV-0.01	734542	672552	61989	****	<0.0001
MSC-sEV-1 vs. HDF-sEV-0.1	734542	669409	65132	****	<0.0001
MSC-sEV-1 vs. HDF-sEV-1	734542	687378	47164	**	0.0018
MSC-sEV-1 vs. HDF-sEV-30	734542	700293	34249	ns	0.0804
MSC-sEV-30 vs. HDF-sEV-0.01	728205	672552	55653	****	<0.0001
MSC-sEV-30 vs. HDF-sEV-0.1	728205	669409	58796	****	<0.0001
MSC-sEV-30 vs. HDF-sEV-1	728205	687378	40828	*	0.0140
MSC-sEV-30 vs. HDF-sEV-30	728205	700293	27913	ns	0.2860
HDF-sEV-0.01 vs. HDF-sEV-0.1	672552	669409	3143	ns	>0.9999
HDF-sEV-0.01 vs. HDF-sEV-1	672552	687378	-14826	ns	0.9386
HDF-sEV-0.01 vs. HDF-sEV-30	672552	700293	-27740	ns	0.2942
HDF-sEV-0.1 vs. HDF-sEV-1	669409	687378	-17969	ns	0.8341
HDF-sEV-0.1 vs. HDF-sEV-30	669409	700293	-30883	ns	0.1665
HDF-sEV-1 vs. HDF-sEV-30	687378	700293	-12915	ns	0.9727

Supplementary Table 7 GLM ANOVA results for proliferation (Folds of OD450 at Day 0) of NHDFs in response to factors of treatment time (Day), cell source of secretome (Cell Source) and secretome fraction type (Treatment) at concentration ≤ 30 μg/mL. DF is the abbreviation for Degree of Freedom.

Variable	DF	F Value	P Value
Day	2	407.25	<0.001
Cell source	1	0.82	0.364
Treatment	2	3.10	0.046
Day*Cell source	2	0.23	0.796
Day*Treatment	4	1.29	0.273
Cell source*Treatment	2	0.34	0.709

Supplementary Table 8 Results of Tukey's post-hoc multiple comparisons between different secretome fraction types at different concentrations (e.g. MSC-sEV-1 means the group treated with MSC-sEV at 1 μg/mL) for their effects on proliferation behaviour of NHDFs. Mean Diff. is the abbreviation for Mean Difference.

Tukey's multiple comparisons test	Mean 1	Mean2	Mean Diff.	Significance	P Value
Blank (DMEM) vs. MSC-sEV-1	1.838	2.101	-0.263	ns	0.9957
Blank (DMEM) vs. MSC-sEV-30	1.838	2.461	-0.624	ns	0.4988
Blank (DMEM) vs. MSC-CM-1	1.838	2.486	-0.648	ns	0.4419
Blank (DMEM) vs. MSC-CM-30	1.838	2.754	-0.917	ns	0.0750
Blank (DMEM) vs. MSC-CM-750	1.838	4.547	-2.710	****	<0.0001
Blank (DMEM) vs. MSC-CM-1500	1.838	4.510	-2.672	****	<0.0001
Blank (DMEM) vs. MSC-NsEV-1	1.838	2.465	-0.627	ns	0.4907
Blank (DMEM) vs. MSC-NsEV-30	1.838	2.527	-0.689	ns	0.3565
Blank (DMEM) vs. MSC-NsEV-750	1.838	3.993	-2.156	****	<0.0001
Blank (DMEM) vs. MSC-NsEV-1500	1.838	4.142	-2.304	****	<0.0001
Blank (DMEM) vs. HDF-sEV-1	1.838	2.310	-0.472	ns	0.8516
Blank (DMEM) vs. HDF-sEV-30	1.838	2.145	-0.307	ns	0.9942
Blank (DMEM) vs. HDF-CM-1	1.838	2.455	-0.617	ns	0.5144
Blank (DMEM) vs. HDF-CM-30	1.838	2.771	-0.933	ns	0.0655
Blank (DMEM) vs. HDF-CM-750	1.838	3.901	-2.063	****	<0.0001
Blank (DMEM) vs. HDF-CM-1500	1.838	4.473	-2.636	****	<0.0001
Blank (DMEM) vs. HDF-NsEV-1	1.838	2.173	-0.335	ns	0.9878
Blank (DMEM) vs. HDF-NsEV-30	1.838	2.382	-0.545	ns	0.6916
Blank (DMEM) vs. HDF-NsEV-750	1.838	3.767	-1.930	****	<0.0001
Blank (DMEM) vs. HDF-NsEV-1500	1.838	4.302	-2.464	****	<0.0001
